# Supplementary material for: Resolving the full spectrum of human genome variation using Linked-Reads
Source: Genome Res. 2019 Apr;29(4):635–45. doi: 10.1101/gr.234443.118 (PMC6442396; doi:10.1101/gr.234443.118)
Supplement: Supplemental Material [file supp_gr.234443.118_Supplemental_longranger-2.2.zip › longranger-2.2.tar.bz2!/lr2.2-public/lib/stan/cmdstan/stan/doc/api/html/math_2functions_2log1p_8hpp_source.html]

Stan: src/stan/math/functions/log1p.hpp Source File


|  |  |
| --- | --- |
|  | Stan  2.5.0  probability, sampling & optimization |


- Main Page
- Related Pages
- Namespaces
- Classes
- Files

- File List
- File Members

All Classes Namespaces Files Functions Variables Typedefs Enumerations Enumerator Friends Macros Pages

- src
- stan
- math
- functions

log1p.hpp

Go to the documentation of this file.

1 #ifndef STAN\_\_MATH\_\_FUNCTIONS\_\_LOG1P\_HPP

2 #define STAN\_\_MATH\_\_FUNCTIONS\_\_LOG1P\_HPP

3

4 #include <limits>

5 #include <boost/math/tools/promotion.hpp>

6

7 namespace stan {

8  namespace math {

9

37  template <typename T>

38  inline typename boost::math::tools::promote\_args<T>::type

39  log1p(const T x) {

40  using std::log;

41  if (!(x >= -1.0))

42  return std::numeric\_limits<double>::quiet\_NaN();

43

44  if (x > 1e-9 || x < -1e-9)

45  return log(1.0 + x); // direct, if distant from 1

46  else if (x > 1e-16 || x < -1e-16)

47  return x - 0.5 \* x \* x; // 2nd order Taylor, if close to 1

48  else

49  return x; // 1st order Taylor, if very close to 1

50  }

51

52  }

53 }

54

55 #endif

stan::math::log1p

boost::math::tools::promote\_args< T >::type log1p(const T x)

Return the natural logarithm of one plus the specified value.

**Definition:** log1p.hpp:39

stan::math::log

Eigen::Matrix< T, Rows, Cols > log(const Eigen::Matrix< T, Rows, Cols > &m)

Return the element-wise logarithm of the matrix or vector.

**Definition:** log.hpp:16

stan::math::e

double e()

Return the base of the natural logarithm.

**Definition:** constants.hpp:86

stan::agrad::log

fvar< T > log(const fvar< T > &x)

**Definition:** log.hpp:15


---

[ Stan Home Page ]

*© 2011–2014,
Stan Development Team.*
